# Factors influencing physical functional status in intensive care unit survivors two years after discharge

**DOI:** 10.1186/1471-2253-13-11

**Published:** 2013-06-18

**Authors:** Jaqueline S Haas, Cassiano Teixeira, Claudia R Cabral, Alessandra H D Fleig, Ana Paula R Freitas, Erika C Treptow, Márcia IB Rizzotto, André S Machado, Patrícia C Balzano, Márcio P Hetzel, Daniele M Dallegrave, Roselaine P Oliveira, Augusto Savi, Silvia RR Vieira

**Affiliations:** 1 Postgraduate Program in Medical Science, Medical School, Universidade Federal do Rio Grande do Sul (UFRGS), Porto Alegre, Brazil; 2 Medical School - Universidade Federal de Ciências da Saúde de Porto Alegre (UFCSPA), Porto Alegre, Brazil; 3 Department of Critical Care – Central-ICU of Complexo, Hospitalar da Santa Casa, Porto Alegre, Brazil; 4 Department of Critical Care, Hospital Moinhos de Vento, Porto Alegre, Brazil

**Keywords:** Activities of Daily Living, Physical Functional Status, Intensive Care Unit, Long-term Care, Mortality, Prognosis, Health-related Quality of Life

## Abstract

**Background:**

Studies suggest that in patients admitted to intensive care units (ICU), physical functional status (PFS) improves over time, but does not return to the same level as before ICU admission. The goal of this study was to assess physical functional status two years after discharge from an ICU and to determine factors influencing physical status in this population.

**Methods:**

The study reviewed all patients admitted to two non-trauma ICUs during a one-year period and included patients with age ≥ 18 yrs, ICU stay ≥ 24 h, and who were alive 24 months after ICU discharge. To assess PFS, Karnofsky Performance Status Scale scores and Lawton-Instrumental Activities of Daily Living (IADL) scores at ICU admission (K-ICU and L-ICU) were compared to the scores at the end of 24 months (K-24mo and L-24mo). Data at 24 months were obtained through telephone interviews.

**Results:**

A total of 1,216 patients were eligible for the study. Twenty-four months after ICU discharge, 499 (41.6%) were alive, agreed to answer the interview, and had all hospital data available. PFS (K-ICU: 86.6 ± 13.8 vs. K-24mo: 77.1 ± 19.6, p < 0.001) and IADL (L-ICU: 27.0 ± 11.7 vs. L-24mo: 22.5 ± 11.5, p < 0.001) declined in patients with medical and unplanned surgical admissions. Most strikingly, the level of dependency increased in neurological patients (K-ICU: 86 ± 12 vs. K-24mo: 64 ± 21, relative risk [RR] 2.6, 95% CI, 1.8–3.6, p < 0.001) and trauma patients (K-ICU: 99 ± 2 vs. K-24mo: 83 ± 21, RR 2.7, 95% CI, 1.6–4.6, p < 0.001). The largest reduction in the ability to perform ADL occurred in neurological patients (L-ICU: 27 ± 7 vs. L-24mo: 15 ± 12, RR 3.3, 95% CI, 2.3–4.6 p < 0.001), trauma patients (L-ICU: 32 ± 0 vs. L-24mo: 25 ± 11, RR 2.8, 95% CI, 1.5–5.1, p < 0.001), patients aged ≥ 65 years (RR 1.4, 95% CI, 1.07–1.86, p = 0.01) and those who received mechanical ventilation for ≥ 8 days (RR 1.48, 95% CI, 1.02–2.15, p = 0.03).

**Conclusions:**

Twenty-four months after ICU discharge, PFS was significantly poorer in patients with neurological injury, trauma, age ≥ 65 tears, and mechanical ventilation ≥ 8 days. Future studies should focus on the relationship between PFS and health-related quality of life in this population.

## Background

Intensive care unit (ICU) mortality has fallen dramatically since 1980, at a yearly rate of 2.0%. Such reduction can be attributed to changes in the delivery of critical care, including increased capacity, establishment of clinical networks and outreach services, implementation of ventilator care bundles [1], and enhancement of strategies to improve decision-making [2-4] and communication among staff, patients, and family members [3,5-7].

Nevertheless, ICU survivors are more susceptible to chronic illness [3,8-14] and increased long-term mortality [10-12,15-19]. The cumulative 12-month mortality of ICU survivors has been reported to range between 35% and 43% [20]. Five-year survival following ICU discharge is also lower in these patients when compared to populations matched by age [10,11,19], pre-existing diseases [12], and sex [15,21]; physical function and quality of life may also be compromised as a result of critical illness. Therefore, in addition to short-term outcomes such as hospital mortality, other aspects must be investigated when dealing with life after ICU discharge [22,23].

The goal of this study was to assess physical functional status two years after discharge from an ICU and to determine factors influencing physical status in this population.

## Methods

### Design

This is a prospective cross-sectional cohort study. The medical records of two no-trauma ICUs were reviewed in search of adult patients with an ICU stay ≥24 between December 1, 2005 and December 1, 2006. Twenty-four months after discharge, all survivors were contacted by phone. All living patients who agreed to participate were included in the study.

### Study population

The study included patients from two ICUs: a 31-bed, closed ICU in a private hospital; and an 18-bed, open ICU in a university hospital. Patients with ICU stay < 24 hours were excluded. If the patient was readmitted to the ICU during the year in review, only data relating to the first admission were considered. The number of readmissions was measured only for sample characterization.

### Assessment of PFS

PFS was assessed by the Karnofsky Performance Status Scale, which emphasizes physical performance and dependency. As a means to monitor the results obtained with the Karnosfky scale (since it is expected that poor physical status will entail decreased ability to perform ADLs), the Lawton-Instrumental Activities of Daily Living (IADL) Scale [23-26] was also applied. Karnofsky and Lawton scores were determined during ICU admission (K-ICU and L-ICU) and after 24 months (K-24mo and L-24mo).

### Data collection

Daily records made by ICU physicians and fellows during the admission were reviewed. The following data were collected: baseline characteristics, age, gender, body mass index (BMI), pre-existing diseases, type of admission (medical, planned surgery or unplanned surgery), acute physiologic and chronic health evaluation (APACHE II) score at 1st ICU-day, and diagnosis on ICU admission. K-ICU score, L-ICU score, data on respiratory, cardiovascular, and dialysis treatments, diagnosis of sepsis, days on mechanical ventilation (MV), ICU-length of stay (LOS), sequential organ failure assessment (SOFA), and therapeutic intervention score (TISS) were also retrieved from the medical charts.

Two years after ICU discharge, patients were contacted via telephone. If the patient was unable to answer the telephone interview, the questions were answered by a proxy, if possible the same person providing information during the ICU admission. Proxies were defined as people with daily contact with the patient since before admission to the ICU.

The interviews were performed by one physician and six nurses. They were trained to use the study instruments and participated in a pilot study with 100 patients [6]. Periodic evaluations were performed to determine inter-rater reliability and to make sure that the quality of interviews remained similar/high among data collectors.

In addition to the Karnosfky and Lawton-IADL scales, the telephone interview focused on the patient’s self-perception about their quality of life (a subjective question to test the reliability of the scales used).

### Data classification

Changes in physical performance (K-ICU vs. K-24mo) and ability to perform daily activities (L-ICU vs. L-24mo) were categorized as: (a) functional improvement – increase in at least one of the scores over 24 months; (b) preserved functional status – no change in scores; (c) moderate functional impairment – reduction of up to 29 points in K-24mo vs. K-ICU and reduction of up to 11 points in L-24mo vs. L-ICU; and (d) major functional impairment – reduction ≥ 30 points in K-24mo vs. K-ICU, and ≥ 12 points in L-24mo vs. L-ICU.

As a final question, patients were asked to rate their PFS at 24 months as compared to PFS during ICU-stay (worse, equal, or better).

### Ethics

The study was approved by the ethics committees at Hospital Moinhos de Vento and Complexo Hospitalar da Santa Casa de Porto Alegre. Oral consent was provided at the start of the telephone interview.

### Statistical analysis

Categorical variables were reported as percentages, and between-group comparisons were made by chi-square test. Continuous variables were reported as means ± SD when variables were normally distributed or as medians and interquartile [P_25_-P_75_] range when variables were not normally distributed. Differences between group means were compared by Student *t* test for normally distributed variables. Chi-square tests or Fisher exact test were used to compare group medians for non-normally distributed variables.

A Kaplan-Meier curve was built to show patient survival. Adjusted residuals were used to evaluate associations between categorical variables and outcome. A paired Student *t* test for quantitative variables was used to evaluate pre and post ICU scores. ANOVA followed by the Tukey test evaluated the association of APACHE II with four categories of the Karnofsky and Lawton-ADL indices. To examine the association of the SOFA score with the scale categories, the Kruskal-Wallis test was used, followed by the Tukey test. The analysis was adjusted for BMI, days on MV, type of ICU admission, age, pre-existing diseases, Glasgow coma score, and SOFA. In multivariate analyses, the predictors of functional status decline were expressed as relative risk (RR).

The analyses were performed with SPSS 16.0 software (SPSS Inc., Chicago, IL, USA), and level of significance was set at *p* <0.05.

## Results

### Study population

**Figure 1:**
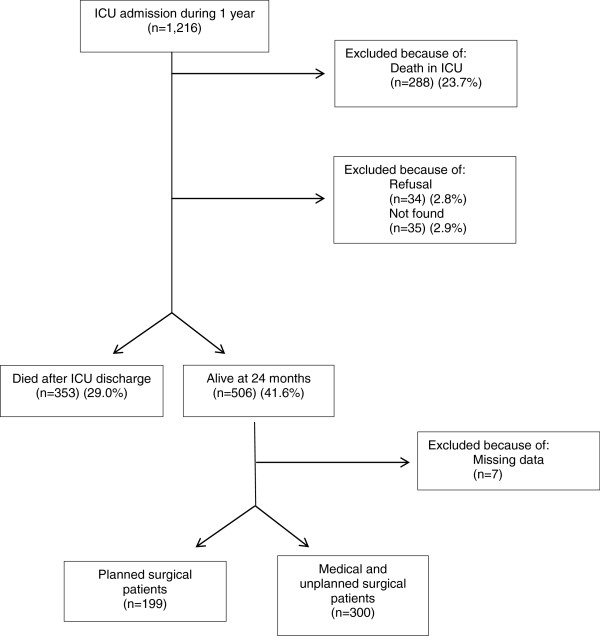
Enrolment scheme.

**Figure 2:**
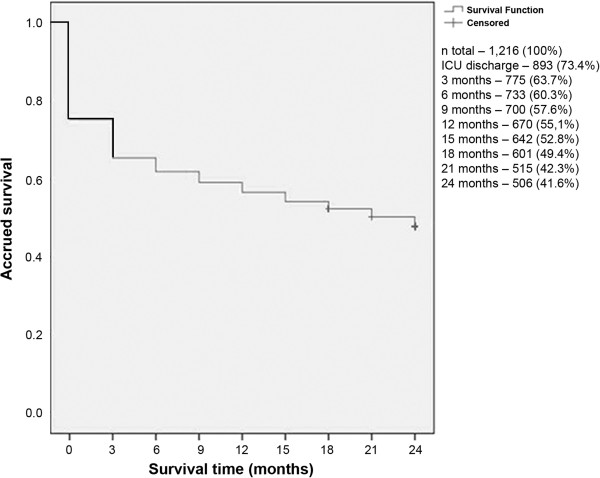
Kaplan-Meier survival curves.

We identified 1,216 patients with ICU stay ≥ 48 hours. Of these, 288 (23.7%) died in the ICU. Phone calls were made to 928 ICU survivors. Thirty-five out of 1,216 patients (2.9%) were not located (lost to follow-up), 34 (2.8%) refused to participate, and 353 had died at the time the call was made: 733 (60.3%) were alive after 6 months, 670 (55.1%) after 12 months, and 601 (49.4%) were alive after 18 months of admission. Five hundred and six patients (41.6%) entered the study (Figure 1 and Figure 2). Hospital data were missing in 7 patients and therefore the results refer to 499 patients with complete hospital records who agreed to participate.

One hundred and eighty-five interviews were answered by the patients themselves, and 314 interviews were answered by proxies (179 [57%] were sons and daughters, 88 [28%] were spouses, and 47 [15%] were caregivers). The ICU and 24-month interviews were answered by the same person in 318 (63%) cases. Therefore, in 181 admissions, the person providing information in the ICU was not the same as the person providing information at 24 months.

The clinical characteristics of the population interviewed 24 months after ICU discharge were as follows: mean APACHE II score of 12.5 ± 7.4, planned surgery (39.9%) as the most frequent type of ICU admission, and predominance of males (51.2%). Differences between patients with planned surgical ICU admission vs. medical plus unplanned surgical ICU admission are shown in Table 1.

**Table 1:** Characteristics of ICU survivors two years after discharge

**ICU admission data**	**Planned surgical admission (n = 199)**	**Medical and unplanned surgical admission (n = 300)**	***p***
Age, years	58.5 ± 18.5	62.4 ± 17.0	*0.01*
Male gender	108 (54.3)	149 (49.7)	*ns*
Number of pre-existing disorders			
≤ 2	176 (88.5)	244 (81.3)	*ns*
> 2	23 (11.5)	56 (18.7)	*ns*
BMI^*a*^			
< 25 Kg/m^2^	81 (40.7)	121 (40.3)	*ns*
≥ 25 Kg/m^2^	118 (59.3)	179 (59.7)	*ns*
Reason for ICU-admission			*ns*
Planned surgery	199	--	
Heart	--	113 (37.7)	
Neurological	--	59 (19.7)	
Respiratory	--	60 (20.0)	
Trauma	--	18 (6.0)	
Other	--	50 (16.6)	
Days on MV			*<0.001*
Did not receive MV	121 (60.8)	222 (74.0)	
1 day	18 (9.1)	59 (19.7)	
2 to 7 days	31 (15.6)	10 (3.3)	
≥ 8 days	29 (14.5)	9 (3.0)	
ICU stay (days)	5 [3:8]	6 [3:11]	
Severity scores			
APACHE II at 1st ICU day	10.2 ± 5.4	14.2 ± 8.2	*<0.001*
SOFA at 1st ICU day	0	[0:1]	*ns*
TISS at 1st ICU day	18 ± 6.5	17.6 ± 6.8	*0.06*
TISS at 3rd ICU day	20.4 ± 6.4	17.4 ± 6.8	*0.02*
TISS discharge	11.5 ± 3.7	10.5 ± 4.7	*0.01*
Evaluation of functional status at ICU admission			
Karnofsky scale (K-ICU)	87.8 ± 10.5	86.6 ± 13.8	*ns*
Lawton scale (L-ICU)	28.8 ± 6.9	27.3 ± 8.6	*ns*
Evaluation of functional status after 24 mo			
Karnofsky scale (K-24mo)	86.0 ± 13.6	77.1 ± 19.6	*<0.001*
Lawton scale (L-24mo)	27.0 ± 11.7	22.5 ± 11.5	*<0.001*

Data are number (%), mean ± standard deviation or median [P_25_:P_75_].

*APACHE* II Acute Physiologic and Chronic Health Evaluation, *BMI* Body mass index, *ICU* Intensive Care Unit, *LOS* Length Of Stay, *MV* Mechanical Ventilation, *SOFA* Sequential Organ Failure Assessment, *TISS* Therapeutic Intervention Scoring System.

### Determinants of physical functional status 24 months after ICU discharge2

**Figure 3:**
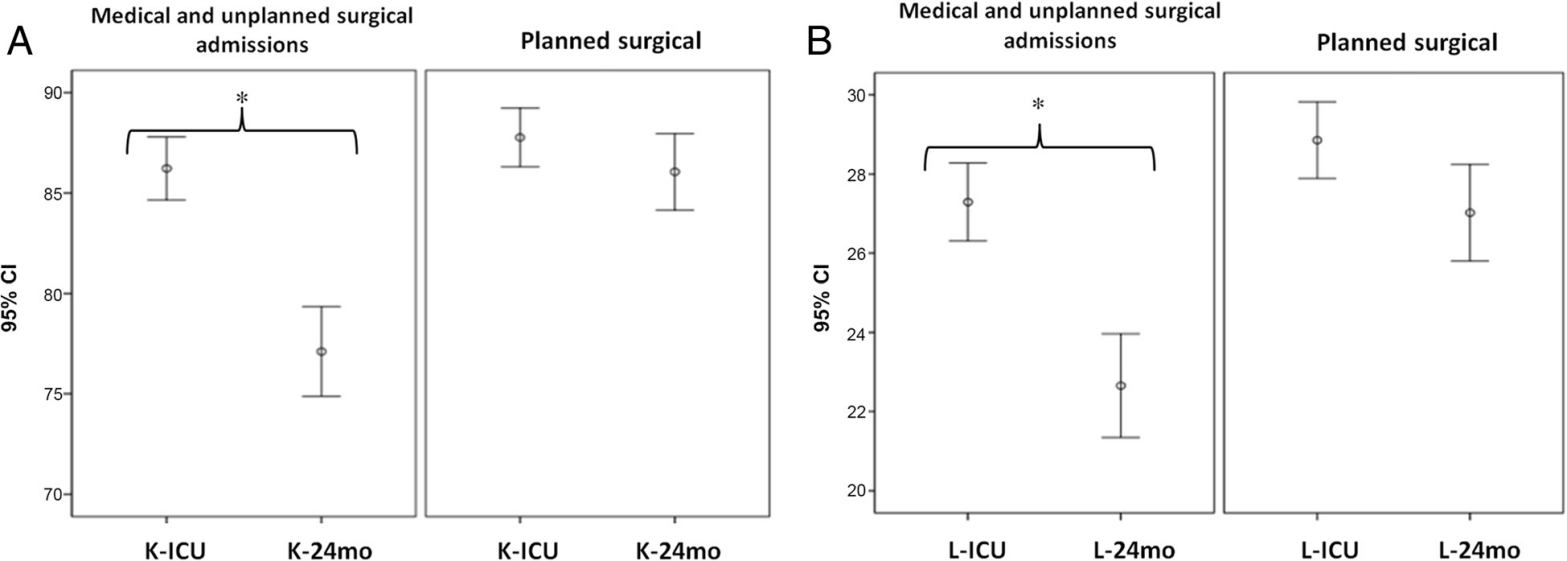
**Karnofsky and Lawton scores. A)** Comparison between mean K scores (K-ICU vs. K-24mo) and **B)** mean L scores (L-ICU vs. L-24mo) in medical and unplanned surgical patients and in planned surgical patients admitted to the ICU.

Both Karnofsky and Lawton scores declined in medical and unplanned surgical patients admitted to the ICU 24 months after discharge (Figure 3B).

The greatest increase in the level of dependency was observed in neurological patients (K-ICU: 86 ± 12 vs. K-24mo: 64 ± 21, RR 2.6, 95% CI, 1.8–3.6, *p* < 0.001) and trauma patients (K-ICU: 99 ± 2 vs. K-24mo: 83 ± 21, RR 2.7, 95% CI, 1.6–4.6, *p* < 0.001) (Table 2 and Table 3). The largest reduction in the ability to perform ADL occurred in neurological patients (L-ICU: 27 ± 7 vs. L-24mo: 15 ± 12, RR 3.3, 95% CI, 2.3–4.6 *p* < 0.001), trauma patients (L-ICU: 32 ± 0 vs. L-24mo: 25 ± 11, RR 2.8, 95% CI, 1.5–5.1, *p* < 0.001), age ≥ 65 years (RR 1.4, 95% CI, 1.07–1.86, *p* = 0.01) and patients receiving MV for more than eight days (RR 1.48, 95% CI, 1.02–2.15, *p* = 0.03) (Table 2 and Table 3).

**Table 2:** Percentage of medical and unplanned surgical patients who had their ability to perform activities of daily life affected after ICU admission according to the Karnofsky and Lawton-instrumental activities of daily living scales

**Variables**	**n**	**Functional impairment (%)**				***p***
		**Severe**	**Moderate**	**No change**	**Improvement**	
Age (Karnofsky), years						*<0.001*
< 65	*265*	7	26	53	14^*a*^	
≥ 65	*234*	13^*a*^	24	61	2	
Age (Lawton)						*<0.001*
< 65	*265*	*12*	14	66^a^	8^a^	
≥ 65	*234*	*20*^*a*^	23^a^	57	0	
Stay in ICU (Karnofsky)						*<0.001*
< 7 days	*401*	7	24	59^*a*^	10^*a*^	
8-14 days	*52*	23^*a*^	29	48	0	
≥ 15 days	*46*	23^*a*^	30	42	5	
Stay in ICU (Lawton)						
< 7 days	*401*	13	17	65^a^	5	
8-14 days	*52*	21	25	54	0	
≥ 15 days	*46*	35^a^	16	40	9	
BMI^*a*^ (Karnofsky)						*<0.001*
< 25 Kg/m^2^	*202*	11	26	58	5	
≥ 25 Kg/m^2^	*297*	4	21	51	24^*a*^	
BMI^*a*^ (Lawton)						*<0.001*
< 30 Kg/m^2^	*202*	17	18	63	2	
≥ 30 Kg/m^2^	*297*	10	18	55	17^a^	
Reason for ICU admission (Karnofsky)						*<0.001*
Heart	*113*	5	22	69^*a*^	4	
Neurological	*59*	39^*a*^	34	27	0	
Respiratory	*60*	8	23	69^*a*^	0	
Trauma	*18*	22	45^*a*^	33	0	
Planned surgery	*199*	3	21	57	19^*a*^	
Other	*50*	12	30	54	4	
Reason for ICU admission (Lawton)						*<0.001*
Heart	*113*	11	13	73^a^	3	
Neurological	*59*	51^a^	30^a^	17	2	
Respiratory	*60*	8	20	70	2	
Trauma	*18*	33	17	50	0	
Planned surgery	*199*	8	18	66	8^a^	
Other	*50*	20	14	62	4	
Days on MV (Karnofsky)						*<0.001*
Did not use MV	*352*	9	27^*a*^	55	9	
1 day	*78*	5	17	67	11	
2 to 7 days	*37*	13	16	68	3	
≥ 8 days	*32*	31^*a*^	25	44	0	
Days on MV (Lawton)						*0.021*
Did not use MV	*352*	*15*	19	61	5	
1 day	*78*	*10*	12	73^a^	5	
2 to 7 days	*37*	*19*	13	65	3	
≥ 8 days	*32*	*33*^*a*^	26	38	3	
APACHE II at 1st ICU day	*499*	14 ± 6^§^	12 ± 6.5^§*^	13 ± 8^§^	8 ± 5^*^	0.02^*^
SOFA at 1st ICU day	*499*	(0 0:2)^§^	(0 0:1)^§*^	(0 0:0)^*^	(0 0:0)^*^	*0.003*^*b*^
Subjective impression (Karnofsky)						*<0.001*
Equal	*172*	1	17	78^*a*^	4	
Worse	*156*	30^*a*^	53^*a*^	17	0	
Better	*171*	0	7	72^*a*^	21^*a*^	
Subjective impression (Lawton)						*<0.001*
Equal	*172*	4	14	82^a^	0	
Worse	*156*	46^a^	30^a^	24	0	
Better	*171*	1	11	75^a^	12^a^	

^*a*^ Adjusted residuals > 1.96;

^*b*^*p* value according to Kruskal-Wallis;

^§, *^ Equal symbols indicate absence of statistically significant difference according to Tukey test.

*APACHE* II Acute Physiologic and Chronic Health Evaluation, *BMI* Body Mass Index, *ICU* Intensive Care Unit, *MV* Mechanical ventilation, *SOFA* Sequential Organ Failure Assessment.

**Table 3:** Probability of decrease in physical functional score 24 months after ICU discharge

**Characteristic**	**RR**	**95% CI**	***p***
Reason for ICU admission			
Medical and unplanned surgery (Karnofsky)	1.3	1.1 – 1.5	0.04
Medical and unplanned surgery (Lawton-ADL)	1.3	1.01 – 1.65	0.03
Neurological (Karnofsky)	2.6	1.8 – 3.6	< 0.001
Neurological (Lawton-ADL)	3.3	2.3 – 4.6	< 0.001
Trauma (Karnofsky)	2.7	1.6 – 4.6	< 0.001
Trauma (Lawton-ADL)	2.8	1.5 – 5.1	0.001
Age ≥ 65 years (Lawton-ADL)	1.4	1.07 – 1.86	0.01
MV ≥ 8 days (Lawton-ADL)	1.48	1.02 – 2.15	0.03

*ICU* Intensive Care Unit, *MV* Mechanical Ventilation, *RR* Relative Risk.

### Self-perception of physical functional status

The decline in PFS subjectively perceived by patients was in agreement with the reduction in PFS indices measured. One hundred and fifty-six patients or proxies perceived functional status as worse than during the ICU admission. Of the patients who reported feeling worse, 83% (*p* < 0.001) had moderate to severe functional impairment by Karnofsky index, and 76% (*p* < 0.001) by Lawton-IADL index (Table 2).

## Discussion

In the present study with ICU survivors interviewed 24 months after discharge, we observed a decline in physical functional status as measured by the Karnofsky and Lawton-IADL scales, especially in patients with neurological diagnoses or trauma, age ≥ 65 years or 8 days or more on mechanical ventilation.

Nearly all patients with chronic critical illness leave the hospital with profound impairment of physical function, cognitive status, or both, requiring institutional care [27,28]. A systematic review of the literature showed that functional impairment is closely associated with age and disease severity [29]. After ICU discharge, elderly patients have been observed to require more assistance than younger counterparts to perform tasks such as using public transportation, shopping, and doing the laundry [3,30]. Our study confirmed this loss of ability to perform independently in patients aged ≥ 65 years.

Conversely, whereas elderly patients often had good PFS or perceived their PSF as better than before critical illness, trauma patients, who were usually healthy and young before ICU admission [23], may experience a substantial decline in PFS after the trauma, both in physical and psychosocial dimensions [31]. Delusional memories, depression [32], and the inability to return to work negatively [33] influenced their perceived quality of life. Our study confirmed that trauma decreases the ability to perform activities independently by 2.8 times, and increases the level of dependency by 2.7 times.

The need for prolonged intensive care may also affect prognosis in terms of the ability to perform ADL [34]; previous articles [35,36] have reported that the inability to independently perform activities of daily living is a major factor affecting health-related quality of life (HRQoL) in ICU survivors. In that sense, prolonged MV appears to reduce life quality and expectancy in the long term [34,37,38]. Our study demonstrated that the use of MV for 8 or more days reduced the ability to perform ADL by 1.48 times. According to some authors, 5% to 20% of ICU patients receive MV, and 25% require MV for more than seven days [39]. In our study, 29.5% required MV and 21.8% of these received ventilatory support for more than eight days.

In our paper, patients with medical and unplanned surgical admissions had decreased PFS; however, this finding did not necessarily imply a reduction in the patients’ actual ability to perform ADLs. Orwelius et al. [40,41] suggested that pre-existing disease is the most important factor for long-term HRQoL after critical illness, and not the factors related to ICU-stay. This was not true for our patients, in whom the presence of pre-existing disease had less impact on PFS than prolonged MV and type of ICU admission (neurological and trauma patients).

Many studies refer to quality of life instead of functional status. HRQoL is a broad concept, which encompasses the ability to perform ADL [24]. In this study, two scales were used in the assessment of PFS, so as to increase the reliability of our results. The Karnofsky index (emphasizing the physical performance and dependency) was chosen because it covers more general aspects of the ability to perform ADL and because it is easy to interpret. Functional impairment has a direct impact on HRQoL because it limits autonomy and physical and mental abilities [13]. Future studies should also address other issues in relation to PFS, such as cognitive impairment, sleep disturbances, post-traumatic stress disorder, the rehabilitation process, employment status, and cultural and payment differences, can influence quality of life in a less tangible way than, for example, physical impairments after major trauma.

Studies assessing HRQoL after ICU suggest that ICU patients do not return to the same level of health that they had before they fell ill [23,29], and that their HRQoL is lower than that of the general population, at least in the early years [3,9,13,15,17,18,20]. According to Oeyen et al. [23], a follow-up of 12 or 24 months is probably the best to capture changes that have a negative impact quality of life after intensive care.

The strengths of present study include a large sample (n = 499), the fact that possible seasonal variations were accounted for (all admissions in one-year), and a long follow-up period (two-years), in addition to the combined use of two scales to increase the reliability of results and a low rate of individuals lost to follow-up (2.9%). However, some limitations must also be addressed: (a) the interviews were conducted by phone and not face-to-face with the patients. However, 26 of the 53 authors cited by Oeyen et al. [23] also conducted telephone interviews; (b) only physical functional status, and not HRQoL, was measured; (c) the fact that some interviews were answered by proxies. However, the literature varies concerning the effect of using proxies. Some authors suggest that proxies (next-of-kin) may underestimate quality of life in their relatives [18,23]; (d) finally, the present population included many patients with cardiovascular problems and elective surgery, that is, a group of not very sick patients that may not reflect the usual critical care group of patients. Therefore, the present results may not allow generalization.

## Conclusions

Twenty-four months after ICU discharge, PFS was significantly poorer in patients with neurological injury, trauma, age ≥ 65 tears, and mechanical ventilation > 8 days. Future studies should also focus on the relationship between PFS and HRQoL in this population.

## Abbreviations

ADL: Activity daily living; APACHE: Acute physiological and chronic health evaluation; ARDS: Acute respiratory distress syndrome; ATS: American thoracic society; BMI: Body mass index; COPD: Chronic obstructive pulmonary disease; K: Karnofsky index; GOS: Glasgow outcome score; HRQoL: Heath-related quality of life; ICU: Intensive care medicine; L: Lawton index; LOS: Length of stay; MV: Mechanical ventilation; NYHA: New York Heart Association; PFS: Physical functional status; RR: Relative risk; SOFA: Sequential organ failure assessment; TISS: Therapeutic intervention scoring system.

## Competing interests

The authors declare that they have no competing interests.

## Authors’ contributions

JS Haas and C Teixeira reviewed the literature and wrote the manuscript. JS Haas, C Teixeira, CR Cabral, AHD Fleig, APR Freitas, EC Treptow, and MIB Rizzotto conducted the telephone interview. AS Machado, MP Hetzel, DM Dallegrave, PC Balzano, and RP Oliveira collected the data base of ICU. A Savi and SRR Vieira significantly contributed for the manuscript. JS Haas performed the statistical analysis. JS Haas and C Teixeira assure the accuracy of the presented data. All authors have read and approved the final manuscript.

## Pre-publication history

The pre-publication history for this paper can be accessed here:

http://www.biomedcentral.com/1471-2253/13/11/prepub
